# Impact of Short-Term and Prolonged (Multi-Year) Droughts on Tree Mortality at the Individual Tree and Stand Levels

**DOI:** 10.3390/plants14131904

**Published:** 2025-06-20

**Authors:** Goran Češljar, Zvonimir Baković, Ilija Đorđević, Saša Eremija, Aleksandar Lučić, Ivana Živanović, Bojan Konatar

**Affiliations:** 1 Department of Spatial Regulation, GIS and Forest Policy, Institute of Forestry, Kneza Višeslava 3, 11030 Belgrade, Serbia; ilija.djordjevic@forest.org.rs (I.Đ.);; 2 Department of Forestry and Environmental Protection, Public Enterprise “Srbijašume”, Bulevar Mihajla Pupina 113, 11070 Beograd, Serbia; 3 Department of Forest Establishment, Silviculture and Ecology, Institute of Forestry, Kneza Višeslava 3, 11030 Belgrade, Serbia; 4 Department of Genetics, Plant Breeding, Seed and Nursery Production, Institute of Forestry, Kneza Višeslava 3, 11030 Belgrade, Serbia

**Keywords:** forest mortality, tree mortality, multi-year droughts, short-term droughts, drought stress

## Abstract

Droughts accompanied by high temperatures are becoming increasingly frequent across Europe and globally. Both individual trees and entire forest ecosystems are exposed to drought stress, with prolonged drought periods leading to increased tree mortality. Therefore, continuous monitoring, data collection, and analysis of tree mortality are essential prerequisites for understanding the complex interactions between climate and trees. This study examined the effects of short-term and prolonged (multi-year) droughts on the mortality of individual trees and forests in Serbia. The analysis was based on datasets from our previous research on the influence of drought and drought duration on individual tree mortality in Serbian forest ecosystems, supplemented with new data collected through the International Co-operative Programme on Assessment and Monitoring of Air Pollution Effects on Forests (ICP Forests). Additionally, we incorporated data from the public enterprise (PE) “Srbijašume”, which manages forests in Central Serbia, focusing on random yields resulting from natural disasters (droughts). These data enabled a comparative assessment of the findings on increased mortality and drought impact at both the individual tree level and the stand level. This study identifies key similarities and differences in tree mortality trends based on drought duration and examines their correlations within the same time frame (2004–2023). By analysing climatic conditions across Serbia, we provide evidence of the interaction between drought periods and increased forest mortality, which we further confirmed by calculating the Standardized Precipitation Evapotranspiration Index (SPEI). We also address the tree species that were most sensitive to the effects of drought. Our findings indicate that prolonged (multi-year) droughts, accompanied by high temperatures, have significantly contributed to increased tree mortality over the past decade. Successive multi-year droughts pose a substantial threat to both individual trees and entire forests, producing more severe and persistent responses compared to those caused by single-year droughts, which forests and individual trees are generally more capable of tolerating. Moreover, due to prolonged drought stress, trees weaken, leading to delayed mortality that may manifest several years after the initial drought event. The observed increase in tree mortality has been found to correlate with rising temperatures and the growing frequency of prolonged droughts over the past decade. Especially, intense droughts in the growing season (April–September) have a very negative impact on forests.

## 1. Introduction

Air temperature and precipitation have long been recognised as two of the most critical climatic factors influencing vegetation and its distribution, as well as being essential for the healthy growth and development of trees. If any of these factors fall below the minimum required level—particularly during the growing season—it can induce stress in trees and potentially compromise their survival. The availability of soil moisture also plays a crucial role in how trees respond to drought stress. Consequently, the magnitude of response can be modulated by the soil properties [1], root system depth [2], and species’ drought tolerance [3]. Considering the above, there is a growing focus on the importance of continuous monitoring of tree mortality as a key factor for understanding these processes [4].

The impact of drought on forest ecosystems has been widely explored in the literature [5,6,7,8,9,10,11]. Drought is widely regarded as one of the most damaging natural disasters and a major driver of tree mortality [8]. It can last from several months to multiple years, during which it affects extensive forested areas [12]. While ecosystems such as forests may withstand one-year droughts, repeated or prolonged drought exposure through multi-year drought events can significantly disrupt their functioning [9]. In this context, the present study focuses on drought periods and discusses the effects of both single-year and multi-year droughts on forests.

Over the past decade, a growing body of research has explored the relationship between drought and tree mortality. Some studies suggest that large, mature trees are more susceptible to drought due to their higher water requirements and higher rates of evapotranspiration [7], while others argue that young trees are more vulnerable owing to their shallow root systems and limited access to deep soil moisture [13]. Some studies report that drought-induced stress, in combination with pest outbreaks, increases defoliation and mortality rates [14]. Similarly, some research studies support the hypothesis that drought, when coupled with insect infestations, significantly contributes to widespread increased tree mortality [15,16]. Pathogenic fungi have also been identified as additional tree mortality agents under drought stress [17], as well as site-specific conditions which can significantly contribute to drought vulnerability and tree mortality [18]. Efforts have also been made to detect early signals of drought-induced tree mortality [19,20], and to integrate all the above-stated factors in correlation with forest ecosystems [21]. Finally, several studies have focused solely on drought as the primary agent of tree decline [10,21,22,23]. Despite the diversity of approaches, the conclusion that all of these studies have in common is that drought acts as a driver of tree mortality.

Drought is most commonly associated with the hottest part of the year, particularly summer [24]. Several studies have highlighted the occurrence of severe meteorological droughts during recent summer seasons, such as those in 2003 [25,26], 2010 [27,28], and 2015 [29,30,31]. In addition, several investigations have identified the period from 2018 to 2020 as one of the driest in recent history [32,33,34], while others report record-setting drought years in specific regions [35]. There is a growing consensus in the literature that drought events are increasing in frequency, duration, and severity on a global scale, with significant implications for vegetation dynamics and forest health [36,37]. In Serbia as well, various droughts have been recorded over the past decade, ranging from short-term events to those lasting several consecutive years. These episodes have occurred alongside historically high temperatures and the warmest years on record during the same period [38], providing a valuable context for analysis. In this study, we build upon our previous findings, which demonstrated that defoliation and visible signs of tree increased mortality may be delayed following drought events. This delay was especially pronounced in coniferous species [39], a pattern also noted in other studies [40], but also confirmed to occur in broadleaved species [23].

The impacts of increasingly intense droughts on various tree species are receiving growing attention. Recent studies focusing on drought-sensitive species, such as Norway spruce (*Picea abies* (L.) Karst.), have revealed that this species exhibits significantly higher mortality rates compared to others also affected by drought, such as European beech (*Fagus sylvatica* L.) [3,41]. Moreover, several studies have highlighted that silver fir (*Abies alba* Mill.), in addition to Norway spruce, is showing signs of increasing vulnerability to drought stress [42,43]. In any case, recent findings clearly indicate that mortality rates in both Norway spruce and European beech have risen following years of severe drought [44], while more frequent and intense droughts will continue to have negative impacts on the vitality of key European tree species [3,45].

In this study, we utilised data from permanent observation plots [46] in Serbia, where individual forest tree decline has been systematically monitored. These data were compared with stand-level mortality data (random yield) provided by the PE “Srbijašume”, a forest management organisation in Serbia. This study is based on a well-distributed dataset that encompasses tree mortality at both the individual and stand levels, complemented by long-term climatic records, including precipitation and extreme temperature data, spanning a 20-year period (2004–2023). The characteristics of short-term and prolonged droughts were examined using the Standardised Precipitation Evapotranspiration Index (SPEI), which allowed for the assessment of temporal variability in drought intensity, frequency, and duration. A review of the existing literature revealed no studies that have addressed the impacts of short-term and prolonged droughts on individual trees and groups of trees in this manner. This study was based on the hypothesis that prolonged drought events have a more pronounced influence on the mortality rates of both individual trees and larger tree groups, compared to short-term droughts. To test this hypothesis, we conducted an analysis of climate and tree mortality data spanning the past two decades. The central research question was whether a statistically significant correlation exists between drought duration and increased tree mortality at both the individual and stand levels.

A key limitation of this study was the unavailability of stand-level mortality data prior to 2013, due to the absence of a systematic monitoring framework at that scale in earlier years. The dataset used for the period from 2013 was compiled from the currently available database of the PE “Srbijašume”.

The primary objective of this study was to evaluate the influence of short-term and prolonged (multi-year) drought events on tree mortality at individual tree and forest stand levels. The main aim of this study was addressed through several specific objectives: to identify the years in which short-term and prolonged droughts occurred, along with their intensity, frequency, and duration; to conduct an in-depth analysis of temperature and precipitation trends throughout the whole study period; to examine tree mortality, both at the level of individual trees and larger groups, over the same study period; to provide an overview of the species most vulnerable to drought in the study area and finally to establish correlations between drought events and the mortality of individual trees and tree groups, in order to enhance understanding of the complex interactions between climatic processes and forest trees. Accordingly, the findings of this study offer valuable insights into the growing impacts of drought on individual trees and forest stands.

## 2. Results

### 2.1. Mortality of Individual Trees and Larger Tree Groups

**Figure 1:**
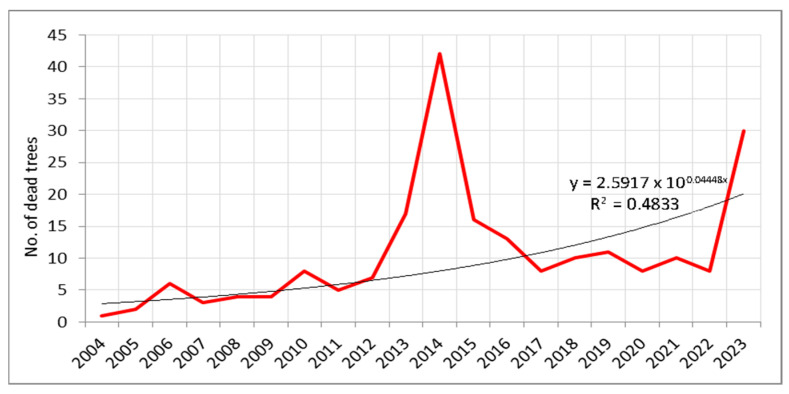
Increased Mortality of Individual Trees on Sample Plots (2004–2023).

**Figure 2:**
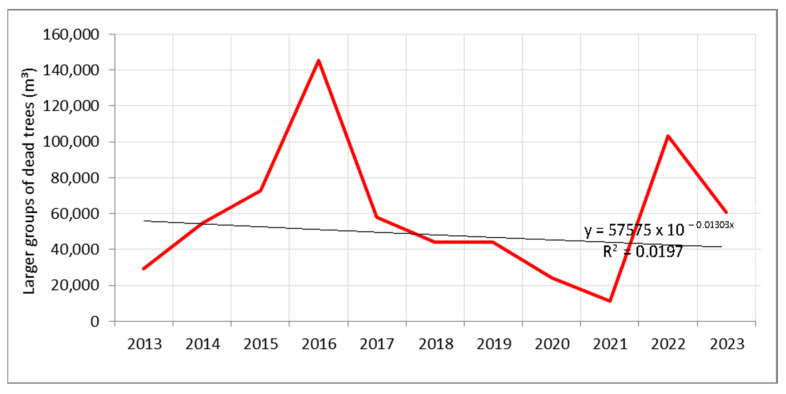
Increased mortality of larger groups of trees (random yields) within Forest Estate (FE) managed by PE “Srbijašume”.

Two distinct periods of increased tree mortality were observed over the course of the study period (Figure 1). The first increase in tree mortality occurred between 2013 and 2016, with the highest number of dead trees recorded in 2014. The second increase in mortality was documented in 2023. In total, 212 trees died during the entire observation period, with 117 of them (55.2%) dying during the two identified mortality peaks—2013–2016 and 2023 (Figure 1 and Supplementary Table S1). Furthermore, a consistent positive exponential increase in the mortality rate of individual trees on the sample plots was observed throughout the study period (Figure 1). In addition to the analysis of individual tree mortality, an assessment of the most vulnerable tree species within the study area was conducted (Supplementary Table S2). Based on the average number of trees recorded during the study period—that is, the relative share of individual species in the total sample—the most vulnerable species during the periods of increased mortality recorded between 2013–2016 and in 2023 (Figure 2) were Norway spruce and silver fir. These two species showed high mortality rates relative to their share in the sample. In addition, broadleaved species such as European beech and oaks (Turkey oak and Hungarian oak), which are also the most widespread species in the study area, showed increased mortality during the same periods. Overall, most of the dominant tree species demonstrated higher mortality rates during these years (Supplementary Table S2).

Similar mortality patterns were observed when analysing larger tree groups over a period twice as short (Figure 2). The first increase in mortality was recorded between 2015 and 2016, with the highest number of dead trees observed in 2016, while the second increase occurred in 2022. According to records of random yield during the study period, a total of 647,492 m^3^ of deadwood was recorded. Of this amount, 321,198 m^3^—or 49.6% of the total volume—was documented during the years of increased mortality (2015–2016 and 2022) (Figure 2 and Supplementary Table S3). Although the exponential growth trend in mortality among larger tree groups over the observation period shows a slight decline, it should be noted that this trend is based on a period twice as short.

Considering the mortality of both individual trees and larger tree groups, it is evident that during the first recorded mortality events in both cases (2013–2016 for individual trees and 2015–2016 for larger groups), the mortality rate increased from year to year. In contrast, the second observed mortality events were more abrupt, occurring suddenly in 2023 for individual trees and in 2022 for larger groups (Figure 1 and Figure 2). Moreover, during these few years of elevated mortality, the total number of dead individual trees and larger tree groups accounted for approximately 50% of all recorded mortality over the entire study period.

### 2.2. Climatic Characteristics Analysis

**Figure 3:**
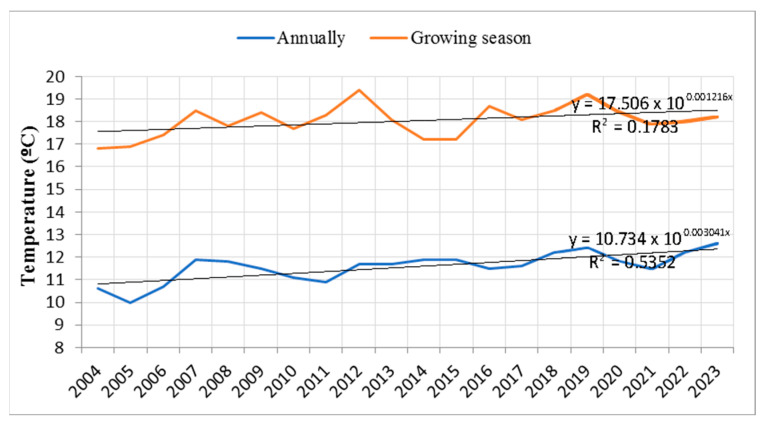
Trends in mean annual air temperature and air temperature during the growing seasons (2004–2023).

The time series analysis of annual air temperatures and air temperatures during the growing season revealed a positive exponential trend in temperature increase over the 2004–2023 period (Figure 3). This trend is particularly evident when comparing mean annual air temperatures in the final years of the observation period to those at the beginning of the study. During the growing seasons, the highest mean annual temperatures were recorded in 2012 and 2019.

**Figure 4:**
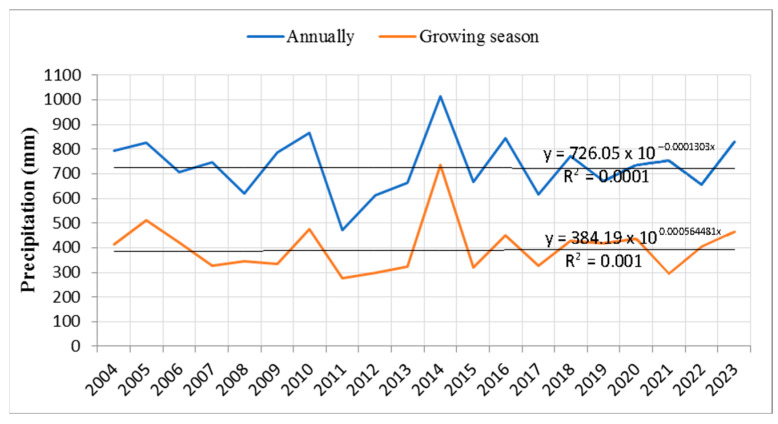
Trend of annual and growing season precipitation sums (2004–2023).

Due to the uneven spatial and temporal distribution of precipitation, the time series analysis of annual and growing season precipitation sums did not reveal a significant positive or negative trend for the period 2004–2023 (Figure 4). Regarding annual precipitation sums, it can be observed that 2011 stood out as the year with the lowest average total precipitation, as did the period from 2011 to 2013. In contrast, precipitation levels in other years varied but remained within a relatively consistent range. It is also evident that in most years, more than 50% of the total annual precipitation occurred during the growing season. Notably low precipitation during the growing season was recorded in 2011 and 2012, while 2021 was marked by an unusually low precipitation sum during the growing season compared to its annual total.

### 2.3. Long-Term Drought Analysis

**Figure 5:**
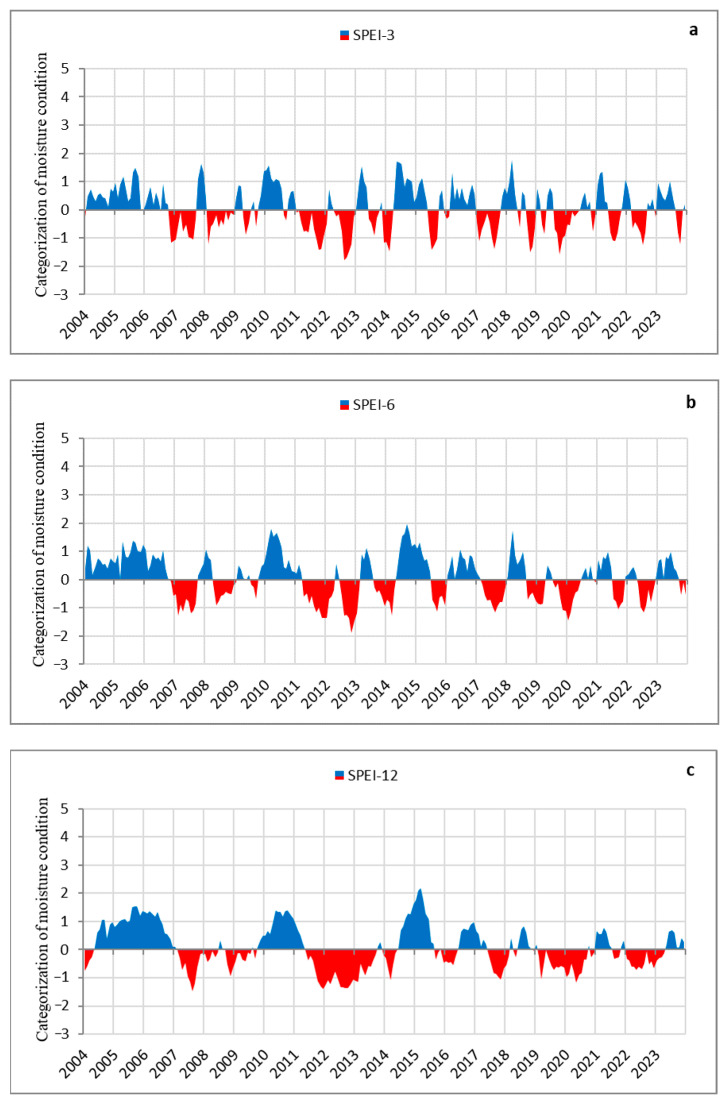
SPEI time series over a region of Serbia: (**a**) SPEI-3, (**b**) SPEI-6, (**c**) SPEI-12.

The results of the SPEI analysis over 3-month (SPEI-3), 6-month (SPEI-6), and 12-month (SPEI-12) intervals, covering the period during which we monitored the mortality of individual trees, as well as more than half of the monitoring period at the stand level, are shown in Figure 5. This analysis particularly emphasises the 12-month timescale (SPEI-12; Figure 5c), as it best illustrates drought trends during the observation period with alternating dry and wet phases, as well as short-term and prolonged droughts. Based on the SPEI-12 analysis, we identified several periods of prolonged and multi-year drought: 2007–2008, 2011–2013, 2019–2020, and 2022–2023. These periods were characterised by consistently negative SPEI values lasting longer than 12 months. Additionally, shorter drought episodes were recorded during 2008–2009 and 2017–2018, with negative SPEI values lasting less than 12 months. The most intense drought occurred between 2011 and 2013, when SPEI remained negative for 29 consecutive months, with some months reaching thresholds for severe drought. In contrast, the negative SPEI-12 values during other prolonged drought periods were not as pronounced and long as those observed in 2011–2013. However, a persistent pattern of prolonged droughts can be observed in recent years (2019–2020 and 2022–2023). The 2019–2020 period stands out slightly more, with 20 consecutive months of negative SPEI values, compared to 16 months during 2022–2023 (Figure 5c). Additionally, when examining SPEI-6 and SPEI-3, we recorded instances of severe drought in certain months (Figure 5a,b). What can be observed over the entire study period is that very few individual years or series of years were entirely without negative SPEI values. The only exception was the initial period of our research (2004–2006) (Figure 5a–c). These findings suggest that the severity of drought episodes has worsened in recent years, both in terms of frequency and duration—marked by high drought occurrence interrupted only by brief wet periods (Figure 5c).

### 2.4. Analysis of Annual Mortality Rates

The results of the statistical analysis of individual tree annual mortality rates, i.e., the mean values of these rates for the observation periods 2004–2008, 2009–2013, 2014–2018, and 2019–2023, are presented in Table 1.

**Figure 6:**
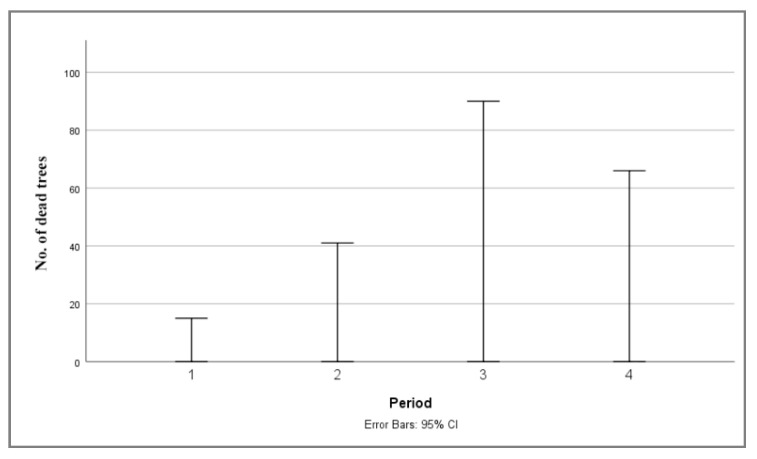
Median plot 95% confidence interval for no. of dead trees monitored in the territory of Serbia in four observation periods: (1) 2004–2008, (2) 2009–2013, (3) 2014–2018, (4) 2019–2023.

Based on the results of the Kruskal–Wallis test for individual tree mortality (Table 2), a statistically significant difference was observed between the test groups. This indicates that there are statistically significant differences in mortality values between the groups (in this case, between the different observation periods). Tree mortality across four periods: (1) 2004–2008, (2) 2009–2013, (3) 2014–2018, and (4) 2019–2023, is shown in Figure 6. A marked increase in the number of trees with 100% defoliation (classified as dead) was recorded during the third period (2014–2018). A slightly lower number of dead trees was recorded in the fourth period (2019–2023), while mortality rates in the first (2004–2008) and second (2009–2013) periods were significantly lower. These results indicate a continuous increase in individual tree mortality from the first to the third period, followed by a decline in the fourth period—although mortality rates remained relatively high.

**Figure 7:**
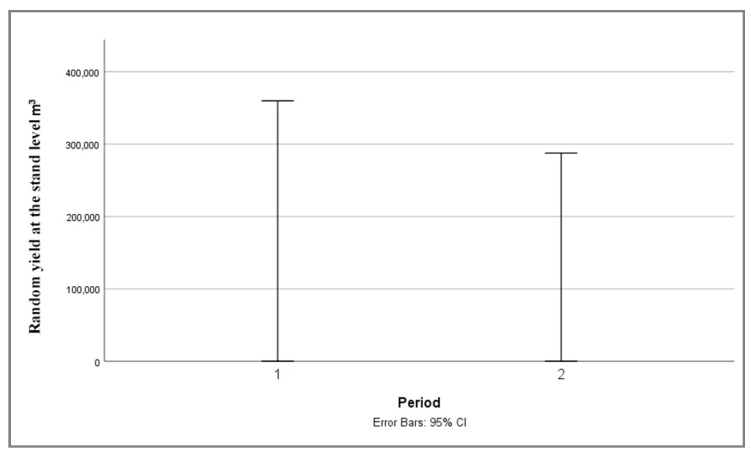
Median plot 95% confidence interval for random yield of larger groups of trees of PE “Srbijašume” in two observation periods: (1) 2014–2018, (2) 2019–2023.

According to the results of the Kruskal–Wallis test for the random yield resulting from the mortality of larger tree groups (Table 3), no statistically significant differences were observed between the study periods. This result suggests that the observed variation across periods can be considered random. However, due to the unavailability of mortality data for larger tree groups prior to 2013, the analysis focuses on the two periods with available data—2014–2018 and 2019–2023. The results indicate a consistent increase in the mortality of larger tree groups (Figure 7), which corresponds to the pattern of individual tree mortality observed over the same periods (Figure 6).

### 2.5. Correlation Between Drought Impact and Mortality of Individual Trees and Larger Tree Groups

**Figure 8:**
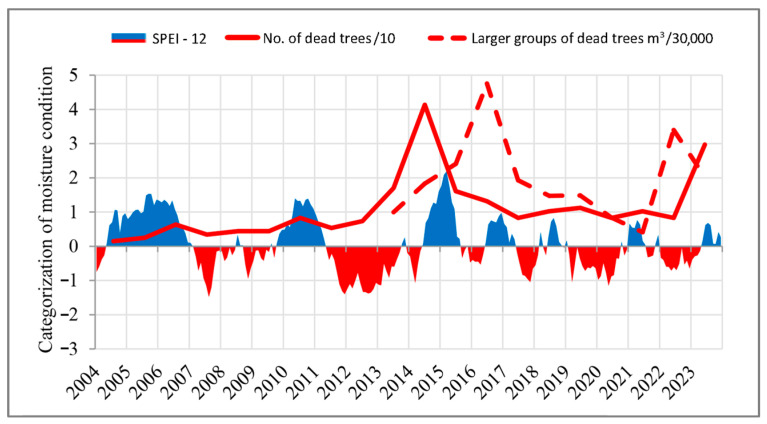
Correlation between SPEI-12 and the mortality of individual trees and larger tree groups.

To gain a more comprehensive understanding of the relationship between drought and the mortality of individual trees and larger tree groups, their analysis is presented in Figure 8. The SPEI-12 was selected as the most appropriate indicator of the intensity, frequency, and duration of droughts during the study period. The results reveal that, following the first recorded prolonged and severe drought episode (2011–2013), a marked increase in mortality was observed first in individual trees (2013–2016), and subsequently in larger tree groups (2015–2016). A similar pattern was evident during the second prolonged drought period (2019–2020 and 2022–2023), with elevated mortality rates recorded for individual trees in 2023 and for larger tree groups in 2022.

## 3. Discussion

It is very difficult to determine how long and severe a drought must be for a tree to die. Numerous interacting factors can influence this process, including tree age, which reflects vitality and ability to withstand stress; tree health status, particularly whether it is already exposed to pathogens that could accelerate its decline; and so on. The literature presents differing theories regarding drought periods, ranging from those suggesting that droughts in the past were longer and more intense than current ones [47] to those questioning whether the severity of the droughts in the last decade has been overestimated [48]. However, the past decade has seen unprecedented increases in temperature and drought duration surpassing all previous extremes since official measurements began, both in Europe [12,27,32,48,49] and globally [50,51]. Furthermore, this trend is clearly observable in our study area as well, which is also confirmed by RHSS data [38].

Our primary objective was to analyse the effects of short-term and prolonged (multi-year) droughts on tree mortality at the levels of individual trees and larger forest complexes (groups of trees) in Serbia. We based our research on the definition that a drought is considered short-term if it results from a precipitation deficit lasting several weeks or months, and long-term if the deficits persist for more than six months [52]. Our analysis, based on the SPEI, identified both types of droughts. The most prominent long-term droughts, in terms of both duration and intensity, occurred during the periods 2007–2008, 2011–2013, 2019–2020, and 2022–2023, while short-term droughts were observed during the transitions from 2008 to 2009 and from 2017 to 2018. These drought periods are consistent with findings at the European level and indicate an increasing frequency of drought events [53], and a trend toward longer and more severe droughts [54], with projections suggesting further intensification in the future [24,55]. Moreover, a growing body of literature over recent years has reported the direct impacts of drought and heatwaves on increased defoliation and mortality in both coniferous and broadleaved species across Europe during the identified drought periods [14,56,57,58,59,60,61,62,63]. According to data published by the European Environment Agency (EEA) [64], Europe experienced the warmest summer and the second warmest year on record, along with the largest overall area affected by drought. Similarly, in Serbia, the available data published by RHSS [38] indicate that recent years rank among the warmest since the beginning of measurements. It emphasised that the occurrence of extreme drought categories has doubled over the last decades in Serbia [65]. These findings collectively suggest that the persistence of droughts and heatwaves, particularly during the growing season, has significantly contributed to tree mortality—as also supported by our research.

Despite the shorter observation period for the mortality of larger tree groups at the stand level compared to individual trees, mortality rates were similarly pronounced and occurred in approximately the same years. Discrepancies in the exact timing of mortality between individual trees and larger groups (random yields) can be partly attributed to differences in data collection methodologies. While data for individual trees were collected once a year during the growing season, observations of larger tree groups were conducted over a longer time frame, spanning the entire year. These differences are particularly evident in coniferous species, as the process of crown die-back can be observed and monitored throughout the entire year.

A similar dynamic of mortality can be observed in the statistical analysis, which shows increased mortality of both individual trees and larger groups during two distinct periods: 2014–2018 and 2019–2023. Based on these findings, it may be assumed that the period for which data on group mortality are unavailable likely corresponds to the mortality data observed in individual trees. Accordingly, random yield data for larger tree groups due to drought prior to 2012 likely had lower values compared to the 2014–2023 period. Climatic characteristics during the two observed periods (2014–2018 and 2019–2023) reveal strong correlations between tree mortality—both individual and grouped—and rising temperatures, low precipitation during the growing season, and the prevalence of prolonged droughts over the past decade. Notably, the rise in mean annual temperatures in recent years suggests that temperatures were considerably above average even during the dormant season. Our findings further indicate that, besides the prolonged drought between 2011 and 2013, two consecutive multi-year droughts—2019–2020 and 2022–2023—had pronounced impacts on forest ecosystems. These periods had the most extreme negative values of the SPEI, in terms of drought intensity, frequency, and duration. Another prolonged drought period (2007–2008) was also identified in our study, though it exhibited less severe SPEI values and did not have the continuity observed in the other periods. Although individual trees responded to some short-term droughts with mortality, our results suggest that trees were generally able to adapt and resist the stress. The exponential increase in the rate of mortality among individual trees was correlated with rising temperatures and the persistent occurrence of extended droughts throughout the study period. In contrast, the exponential trend for larger tree groups showed a negative trend, which may be attributed to the fact that the observation period was twice as short as that for individual trees.

The claim that droughts weaken trees and that their adverse effects can persist for several years after the initial event [66]—i.e., that tree mortality can become evident only several years after a severe drought—was supported by random yield data for larger tree groups presented in our study. For example, the prolonged drought from 2011 to 2013 resulted in a significant increase in deadwood volume only in 2016. A similar delayed response was observed following the prolonged drought of 2019–2020, with a significant rise in deadwood recorded only in 2022. A comparable observation was made for individual dead trees, where large-scale mortality was recorded only after the drought period. Following the prolonged drought in 2011–2013, trees’ increased mortality was recorded in 2014, while the impact of the 2019–2020 drought was not registered until 2023.

Our findings are supported by other studies, which similarly note that in the case of extreme and prolonged drought over a wide area, an increase in tree mortality in the subsequent years can be expected, whereas short-term drought events are generally insufficient to cause high mortality [60]. Moreover, there is a clear trend of forest endangerment due to unfavourable climatic conditions—primarily drought— which compromise trees’ defence mechanisms against insect infestations, making them significantly more susceptible [67]. A review of the literature on this topic identifies Serbia as a potential hotspot for forest mortality based on the observed trends in the occurrence of severe droughts [10]. Additionally, other studies have explicitly highlighted the link between tree mortality and drought in some regions, including Serbia [8,68].

Despite the relatively lower proportion of coniferous species, such as Norway spruce and silver fir, in our overall sample compared to broadleaved species, they demonstrated a greater sensitivity to drought in the study area. Our findings align with other research that indicates these species are significantly impacted by prolonged droughts, even when they grow in the heart of their natural habitat. Similar observations are found in studies conducted in regions with comparable climatic conditions and involving tree species that also occur within our study area [3,42]. European beech, the most common species in the region, has also been shown to struggle during dry spells [69]. This was evident from the increased tree mortality observed during the most severe drought years in our study. A similar trend was noted in other dominant broadleaved species, which, alongside European beech, were notably affected by extended drought conditions [3].

Finally, it is evident that the frequency of high temperatures and increasingly warmer years has been particularly pronounced in Serbia over the past decade [38]. Over the last 20 years, which is the duration of our study, 15 of the hottest years on record in Serbia have been observed, with 9 of the 10 warmest years occurring within the last decade [38]. This trend underscores the significant impact of prolonged and recurrent droughts, combined with elevated temperatures, on large-scale forest increased mortality. Such effects were notably observed following the extended drought of 2011–2013, as well as after two consecutive prolonged drought events in 2019–2020 and 2022–2023, as demonstrated in this study. Moreover, our findings clearly indicate that the ability of forests to resist and recover from drought stress is highly dependent on the intensity, frequency, and duration of drought events—an observation also supported by previous studies [7].

## 4. Materials and Methods

### 4.1. Study Area

**Figure 9:**
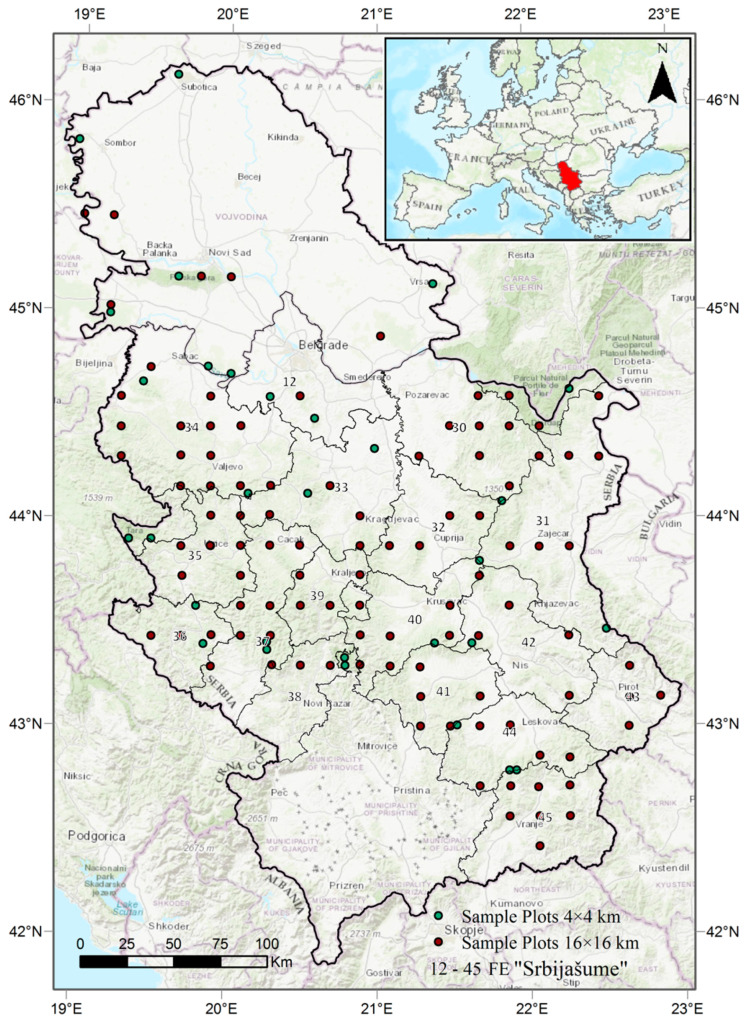
Study area with sample plots (ICP Forests) and Forest Estates (FE) managed by PE “Srbijašume”.

This research was conducted in Central Serbia, within forest areas managed by the public enterprise (PE) “Srbijašume” and 130 permanent sample plots where individual tree monitoring is carried out following the standardised methodology of the International Co-operative Programme on Assessment and Monitoring of Air Pollution Effects on Forests (ICP Forests) [46] (Figure 9). The dominant broadleaved species in the study area include European beech (*Fagus sylvatica* L.), Turkey oak (*Quercus cerris* L.), sessile oak (*Quercus petraea* (Mattuschka) Liebl.), Hungarian oak (*Quercus frainetto* Ten.), and hornbeam (*Carpinus betulus* L.). Among conifers, the most abundant species are Norway spruce (*Picea abies* (L.) Karst.), followed by Austrian pine (*Pinus nigra* Arn.), silver fir (*Abies alba* Mill.), and Scots pine (*Pinus sylvestris* L.) [70]. The monitored tree population included 29 broadleaved and 4 coniferous species. The dominant species account for approximately 84% of the total sample, accurately representing the overall species composition across the study region.

### 4.2. Data Preparation

This study spans a 20-year period of individual tree mortality monitoring, with an average of 2892 trees assessed annually through the ICP Forests network and an 11-year period of monitoring tree mortality at the level of larger tree groups (random yields) conducted over a forest area of 775,772.82 hectares managed by the PE “Srbijašume”.

The dataset on individual tree mortality was initially derived from an internal database covering the period 2004–2018 [39] and was subsequently updated with additional mortality records for the years 2019–2023. These datasets were consolidated to produce a continuous 20-year record of individual tree mortality in Serbia. During data processing, only trees exhibiting 100% defoliation were classified as dead in a given year and included in the analysis. Key variables such as site location, elevation, tree age, species identity, and annual defoliation progression leading up to mortality were also taken into account (Supplementary Tables S1 and S2). Field data collection for individual trees was carried out in the growing season (April–September) every year. In other words, trees were selected based on clear defoliation trends that ultimately resulted in death to facilitate the identification of potential links with drought periods. Finally, to relate the previously collected data on individual tree mortality to short- and long-term drought periods, the analyses also included stand-level data on deadwood volume (random yields) across all management units overseen by the PE “Srbijašume” (Supplementary Table S3). Field data collection for larger groups of trees was carried out throughout the year. Finally, to determine which tree species was most sensitive during drought periods in the study area, we conducted a general analysis of individual dead trees using the mortality database (Supplementary Tables S1 and S2).

### 4.3. Quantification of Climatic Characteristics

The climate of Serbia is classified as temperate continental, exhibiting varying degrees of local features. The spatial distribution of climatic parameters is primarily influenced by geographic location, topography, and local influences resulting from a combination of terrain configuration, large-scale atmospheric pressure patterns, slope exposure, the presence of river systems, vegetation cover, and urbanisation [71]. To evaluate the climatic influence on the study area over the past two decades, mean annual air temperatures and total annual precipitation were calculated using data from 28 main meteorological stations operated by the Republic Hydrometeorological Service of Serbia [38]. In addition to annual values, mean air temperature and total precipitation during the growing season (April–September) were considered particularly relevant for the purposes of this study. Given the large area of study, the data were analysed using a linear regression model with a 95% confidence band.

### 4.4. Drought Index Quantification

To investigate the relationship between drought periods and mortality of both individual trees and larger tree groups, we employed the Standardised Precipitation Evapotranspiration Index (SPEI), following the methodology developed by Vicente-Serrano et al. [72]. To calculate SPEI—which, unlike other indices, incorporates both precipitation and temperature data—we used data obtained from the global SPEI database [73]. SPEI time series over a region were generated for the territory of Serbia using regional coordinates (upper left: 42°25′, 23°25′; lower right: 46°25′, 18°75′). The index was computed for three-month (SPEI-3), six-month (SPEI-6), and twelve-month (SPEI-12) time series which were subsequently analysed to assess drought intensity, frequency, and duration. To evaluate the above-mentioned drought parameters, we focused on the SPEI classification scale, which ranges from extremely wet to extremely dry conditions, as well as the duration of a drought episode, in terms of the number of consecutive months from the moment it starts to its termination. We singled out years in which SPEI values were continuously negative on a monthly scale as they are indicative of drought conditions. Prolonged (multi-year) droughts were defined as those with more than 12 consecutive months or multiple years of negative SPEI values, while short-term droughts were those with fewer than 12 consecutive months of negative SPEI values. In total, we analysed a 20-year period of drought trends in Serbia (2004–2023), focusing on the alternation of wet and dry periods with respect to both frequency and duration (in months).

### 4.5. Statistical Analysis

The statistical analysis at the individual tree level was conducted using data on defoliation trends collected on sample plots, following the methodology outlined in the ICP Forests Manual [74]. Trees exhibiting 100% defoliation were classified as dead, and annual mortality rates were calculated accordingly. Data were then grouped into four observation periods: 2004–2008, 2009–2013, 2014–2018, and 2019–2023. Mortality rates were computed using the formula *m* = 1 − (N_1_/N_0_)^1/t^, where N_0_ is the initial population size and N_1_ is the final population size [75]. Changes in mortality were determined on an annual basis. The obtained data were grouped into multi-year intervals for which average values were computed. For the purpose of calculating the value of *m*, four periods with five-year intervals were defined, as the five-year interval is widely used and recommended by literature sources as an optimal period for maximising comparability across time and study regions [76]. Prior to statistical analysis, data were tested for normality (Supplementary Table S4). Based on the results, non-parametric tests were employed. Specifically, the Kruskal–Wallis test was applied using SPSS statistical software (Version 26, IBM Corp., Armonk, NY, USA).

Available data from the PE “Srbijašume” for the period 2013–2023, concerning larger tree groups (random yields), were used to examine whether they can be related to increased mortality of individual trees during the same period. Following the model used for individual tree mortality data, the analysis focused on larger groups also employed five-year intervals. The main difference in the statistical analysis was related to data availability; therefore, the dataset from PE “Srbijašume” was divided into two five-year periods: the first covering 2014–2018, and the second 2019–2023. For both datasets, the following statistical parameters were calculated: median (M), mean absolute deviation (MAD), minimum (MIN), and maximum (MAX), along with a Kruskal–Wallis test to assess statistical significance.

## 5. Conclusions

Our results demonstrate that prolonged (multi-year) droughts have a substantial impact on tree mortality, affecting both individual trees and larger forested areas, while short-term droughts generally influence only a small number of individual trees. Although trees may exhibit mortality in response to short-term drought events, they are typically capable of adapting to or recovering from such stress. In contrast, prolonged droughts, especially when accompanied by elevated temperatures, significantly increase mortality rates. We also found that the effects of prolonged droughts can persist for several years after the drought period ends. Exponential increases in the mortality of individual trees were correlated with rising temperatures and the recurring occurrence of droughts in the past decade. Furthermore, the persistence of prolonged droughts in recent years (2019–2020 and 2022–2023), accompanied by record-high temperatures, has had a detrimental impact on both individual trees and larger forested areas. This occurred regardless of the drought intensity, which was considerably lower than during the 2011–2013 prolonged drought, a period that also saw widespread tree and forest mortality. These findings suggest that the recurrence and duration of drought stress may be more critical than the intensity alone in driving tree mortality. Particularly detrimental are prolonged droughts during the growing season (April–September), which are strongly associated with forest decline. To get more accurate findings of how drought affects specific tree species in our study area, further research must be conducted. This should involve larger sample sizes with wider forest regions that have experienced heightened mortality following severe droughts.

If current climatic trends continue, a key question remains: will forest trees increasingly lose their capacity to resist extreme heat and drought, or will they gradually adapt over time? Moreover, shifts in seasonal climate patterns—such as sub-zero spring temperatures or high temperatures during winter without precipitation—may further challenge forest resilience and long-term sustainability. To answer these questions, it is essential to continue systematic forest monitoring. Long-term observation will enable a better understanding of the processes of tree mortality caused by drought and high temperatures. Therefore, continuous monitoring is a key tool that can provide reliable long-term data and help identify the main drivers of tree mortality. Lastly, the question of how future climate change will reshape forest ecosystems remains open. It is still uncertain whether forests can adapt rapidly enough to avoid substantial consequences. We therefore expect that our findings contribute to a deeper understanding of prolonged drought dynamics, which are becoming increasingly frequent and severe under ongoing climate change.

## Figures and Tables

**Table 1:** Mean annual mortality rates for individual trees.

Observation Period	Average Mortality Rate
2004–2008	0.0010
2009–2013	0.0029
2014–2018	0.0061
2019–2023	0.0045

**Table 2:** Descriptive and nonparametric statistics for the annual mortality rates of trees monitored in the territory of Serbia for four observation periods. N—Number of samples; M—Median; MAD—Median Absolute Deviation; MIN—Minimum value; MAX—Maximum value.

Period of Observation		N	M	MAD	MIN	MAX	Mean Rank	Test Statistics	Results
2004–2008	1	5	0.0010	0.0005	0.0000	0.0020	3.5	Kruskal–Wallis H	11.749
2009–2013	2	5	0.0025	0.0012	0.0014	0.0061	9.7	df	3
2014–2018	3	5	0.0044	0.0027	0.0027	0.0143	15.4	Asymp. Sig. (*p*-value)	0.008
2019–2023	4	5	0.0034	0.0017	0.0027	0.0101	13.4		

**Table 3:** Descriptive and nonparametric statistics for the random yield at the stand level of PE ”Srbijašume“ for two observation periods. N—Number of samples; M—Median; MAD—Median Absolute Deviation; MIN—Minimum value; MAX—Maximum value.

Period of Observation		N	M	MAD	MIN	MAX	Mean Rank	Test Statistics	Results
2014–2018	1	5	57,955.69	23,641.7	43,927.43	145,168.9	6.4	Kruskal–Wallis H	0.884
2019–2023	2	5	44,208.21	25,746.93	11,092.87	103,279.7	4.6	df	1
								Asymp. Sig.	0.347

## Data Availability

Data are contained within the article and Supplementary Materials.

## Supplementary Materials

The following supporting information can be downloaded at: https://www.mdpi.com/article/10.3390/plants14131904/s1, Table S1: Trends in defoliation on individual trees during the years of research with the final outcome of dying, Table S2: Analysis of the most endangered tree species due to the impact of drought, Table S3: Random yield due to the impact of drought for larger groups of trees within Forest Estates (FE) Managed by Public Enterprise (PE) “Srbijašume”, Table S4: Tests of Normality.
