# Supplementary material for: Impact of Short-Term and Prolonged (Multi-Year) Droughts on Tree Mortality at the Individual Tree and Stand Levels
Source: Plants (Basel). 2025 Jun 20;14(13):1904. doi: 10.3390/plants14131904 (PMC12251964; doi:10.3390/plants14131904)
Supplement: Supplementary file 1 [file plants-14-01904-s001.zip › plants-3669529-supplementary.pdf]

Supplementary Material

Article: **IMPACT OF SHORT-TERM AND PROLONGED (MULTI-YEAR) DROUGHTS ON TREE MORTALITY AT THE INDIVIDUAL TREE AND STAND LEVELS**

This PDF file includes:
Tables S1 to S4

Table S1. Trends in defoliation on individual trees during the years of research with the final outcome of dying.

SP Level I	Locality	Altitude (m)	Stand age	Tree Species	Defoliation (%) per year																			
					2004	2005	2006	2007	2008	2009	2010	2011	2012	2013	2014	2015	2016	2017	2018	2019	2020	2021	2022	2023
52	Kladnica	1389	61-80	*Fagus sylvatica*	10	15	15	10	40	50	15	20	10	10	20	30	70	70	75	50	50	40	50	100
77	Raškovićev zabran	572	uneven aged	*Quercus cerris*	70	55	60	60	50	40	40	50	80	80	10	15	10	15	15	15	15	15	15	100
28	Potaj Čuka	619	61-80	*Fagus sylvatica*	0	0	0	10	10	10	0	10	10	0	0	0	10	10	50	50	40	25	90	100
42	Despotovac	386	uneven aged	*Quercus frainetto*	*									10	0	10	0	0	10	0	0	0	0	100
91	Vlasinsko jezero	1370	21-40	*Betula pendula*	*					10	10	10	10	0	10	10	10	30	70	60	40	20	30	100
74	Nova Varoš	1191	uneven aged	*Picea abies*	10	10	10	10	15	20	20	15	5	0	0	0	10	10	5	0	0	0	0	100
74	Nova Varoš	1191	uneven aged	*Picea abies*	10	10	5	5	5	15	15	10	10	0	0	0	0	0	0	0	0	0	0	100
74	Nova Varoš	1191	uneven aged	*Picea abies*	55	50	25	30	20	10	10	15	15	0	30	10	30	10	0	10	0	0	20	100
74	Nova Varoš	1191	uneven aged	*Picea abies*	50	40	20	20	20	15	15	10	15	10	0	0	10	10	25	0	0	0	0	100
74	Nova Varoš	1191	uneven aged	*Picea abies*	35	35	10	10	10	15	10	15	10	0	0	0	0	0	0	0	0	0	0	100
74	Nova Varoš	1191	uneven aged	*Picea abies*	25	25	15	15	15	20	25	10	5	0	0	0	5	0	0	0	0	0	0	100
74	Nova Varoš	1191	uneven aged	*Picea abies*	25	20	5	5	10	15	15	10	10	0	0	0	10	10	0	0	0	0	0	100
74	Nova Varoš	1191	uneven aged	*Picea abies*	20	20	5	10	15	15	15	15	5	0	0	0	0	0	0	0	0	0	0	100
74	Nova Varoš	1191	uneven aged	*Picea abies*	15	15	10	15	15	15	10	10	10	0	0	0	5	0	15	0	0	0	0	100
37	Vratarnica	231	41-60	*Quercus frainetto*	10	10	60	20	10	20	20	20	10	0	50	15	15	10	15	20	15	25	25	100
415	Maljen	630	61-80	*Abies alba*	10	10	10	10	10	10	0	0	0	10	0	10	10	10	0	0	0	20	65	100
5	Krupanj	575	21-40	*Pinus sylvestris*	*														5	5	0	0	0	100
14	Cer	70	41-60	*Carpinus betulus*	*												10	10	10	10	0	0	0	100
14	Cer	70	41-60	*Acer campestre*	*												10	10	0	10	0	0	0	100
408	Veliki jastrebac	735	81-100	*Fagus sylvatica*	0	0	0	0	0	0	10	10	10	0	0	0	10	40	10	10	20	20	90	100
29	Rudna glava	346	21-40	*Quercus frainetto*	10	10	20	30	10	10	10	10	0	10	20	0	0	10	10	30	30	30	80	100
33	Bukova glava	432	>121	*Quercus petraea*	10	30	10	40	30	0	10	20	20	20	0	80	85	80	80	80	90	90	90	100
101	deliblato	125	81-100	*Robinia pseudoacaccia*	*														80	90	90	95	95	100
101	deliblato	125	81-100	*Pinus nigra*	45	40	35	30	45	30	35	30	20	30	25	15	15	25	30	45	40	50	95	100
101	deliblato	125	81-100	*Pinus nigra*	45	45	50	50	45	50	45	35	35	35	25	30	35	30	65	80	80	70	90	100
423	Kolut Kozara	0	>121	*Quercus robur*	75	75	20	45	80	50	55	50	60	65	50	55	55	50	60	70	70	75	90	100
423	Kolut Kozara	0	>121	*Quercus robur*	*							5	0	20	25	15	25	30	15	20	30	45	80	100
426	Vitonajevačko ostrvo	0	>121	*Fraxinus angustifolia*	10	10	10	20	15	20	10	10	10	15	0	0	5	15	40	40	30	35	55	100
426	Vitonajevačko ostrvo	0	>121	*Fraxinus angustifolia*	*																		40	100
430	Vranić	165	21-40	*Robinia pseudoacaccia*	*										5	40	0	10	0	0	90	95	100	
428	Nemenikuće	279	41-60	*Quercus cerris*	*										30	25	25	15	30	30	40	35	100	
427	Kupinske grede	70	101-120	*Fraxinus angustifolia*	*													25	45	50	40	50	100	
402	Tara II	1151	81-100	*Picea abies*	*												15	15	10	0	0	0	100	
105	Čortanovačka šuma	175	61-80	*Tilia platyphyllos*	20	5	5	10	0	0	0	5	5	5	5	5	0	0	0	0	0	0	100	
32	Severni Kučaj	529	81-100	*Fagus moesiaca*	0	0	10	10	0	0	0	10	20	20	0	40	80	60	80	90	90	90	100	
28	Potaj čuka	619	61-80	*Fagus moesiaca*	*														0	0	0	20	100	
12	Banjani	141	41-60	*Carpinus betulus*	10	10	10	10	10	20	20	10	10	0	10	0	15	0	10	10	0	15	100	
427	Kupinske grede	70	101-120	*Fraxinus angustifolia*	5	10	25	15	15	20	20	20	20	35	15	10	10	40	40	35	40	100		
426	Vitonajevačko ostrvo	0	>121	*Quercus robur*	15	15	20	15	10	10	15	20	10	10	15	45	45	45	25	25	20	100		
424	Hajdučki breg	225	61-80	*Quercus petraea*	45	20	5	40	30	45	35	35	30	30	20	10	15	30	30	30	60	100		
415	Maljen I	630	61-80	*Betula pendula*	*													10	0	0	0	100		
410	Štrbačko korito	344	81-100	*Fagus moesiaca*	15	20	10	10	10	0	0	0	0	0	0	0	15	50	30	80	90	100		
101	Deliblato	125	81-100	*Robinia pseudoacaccia*	70	80	80	80	75	75	70	80	80	85	80	70	75	80	70	80	90	100		
73	Pobijenik	1201	uneven aged	*Picea abies*	15	10	5	10	15	15	5	0	5	10	20	0	25	0	0	0	90	100		
12	Banjani	141	41-60	*Robinia pseudoacaccia*	*								50	0	0	90	80	80	85	85	90	100		
8	Ub	148	41-60	*Quercus frainetto*	20	10	10	10	20	10	10	10	0	0	0	10	0	0	0	0	0	100		
2	Barajevo	293	21-40	*Quercus frainetto*	*								10	0	0	0	0	0	30	15	0	100		
421	Vrsacki breg	370	61-80	*Fraxinus omus*	15	0	30	10	25	25	30	30	30	35	45	50	55	50	30	90	100			
410	Štrbačko korito	344	81-100	*Fagus moesiaca*	15	10	10	10	0	0	10	10	0	5	10	0	15	40	30	40	100			
401	Tara I	1098	101-120	*Abies alba*	15	20	10	10	10	15	0	0	10	0	10	10	15	0	10	85	100			
103	Odžaci	0	<= 20	*Fraxinus excelsior*	*														65	70	100			
40	Svetozarevo	421	41-60	*Fagus sylvatica*	0	0	0	5	0	0	0	0	0	20	25	30	30	20	90	95	100			
33	Bukova Glava	432	>121	*Quercus petraea*	0	40	40	10	20	50	30	20	10	80	0	20	20	60	70	90	100			
32	Severni Kučaj	529	81-100	*Fagus moesiaca*	0	60	60	20	70	50	50	0	10	10	10	50	80	90	90	90	100			
14	Cer	70	41-60	*Acer campestre*	*													0	0	15	100			
429	Smederavska Palanka	114	61-80	*Ulmus minor*	*										0	0	0	0	95	100				
423	Kolut-Kozara	70	>120	*Quercus cerris*	30	25	10	10	5	15	15	5	10	30	35	30	35	30	70	100				
423	Kolut-Kozara	70	>121	*Quercus cerris*	35	30	5	10	20	15	20	30	15	35	40	50	50	70	80	100				
81	Kuršumlija	453	21-40	*Quercus cerris*	20	10	30	20	10	0	10	20	10	80	90	90	90	90	95	100				
73	Pobijenik	1201	uneven aged	*Picea abies*	5	5	5	5	10	10	0	0	0	0	0	0	10	0	95	100				
45	Partizanske Vode	972	61-80	*Pinus sylvestris*	*													0	70	100				
33	Bukova Glava	432	>121	*Quercus petraea*	10	40	10	10	10	10	10	20	20	25	0	20	25	40	90	100				
10	Miličinica	255	61-80	*Quercus frainetto*	10	20	0	10	10	10	0	0	0	0	0	0	0	0	0	100				
2	Barajevo	293	21-40	*Quercus frainetto*	*										20	10	0	0	20	100				
2	Barajevo	293	21-40	*Quercus cerris*	*										10	10	5	0	70	100				
2	Barajevo	293	21-40	*Quercus cerris*	*	20	15	0	20	25	0	5	5	10	0	0	0	0	60	100				
429	Smederavska Palanka	114	61-80	*Ulmus minor*	*										5	0	0	0	100					

SP Level I	Locality	Altitude (m)	Stand age	Tree Species	Defoliation (%) per year																			
					2004	2005	2006	2007	2008	2009	2010	2011	2012	2013	2014	2015	2016	2017	2018	2019	2020	2021	2022	2023
424	Andrvlje Testera	225	61-80	*Quercus petraea*	35	35	35	25	10	15	25	20	35	30	25	15	10	10	100					
421	Vršački Breg	370	61-80	*Quercus petraea*	10	15	20	20	15	30	35	30	30	20	30	25	30	30	100					
415	Maljen	630	61-80	*Fagus sylvatica*	10	30	10	10	0	10	5	0	0	10	0	40	75	80	100					
406	Jamnjaci	1400	81-100	*Picea abies*	30	20	5	0	10	0	0	0	0	0	25	30	10	10	100					
404	Bunatovac	1120	41-60	*Fagus sylvatica*	0	0	0	10	0	20	10	10	5	0	0	0	0	0	100					
404	Bunatovac	1120	41-60	*Fagus sylvatica*	10	5	0	10	0	0	40	15	10	0	10	0	10	30	100					
404	Bunatovac	1120	41-60	*Fagus sylvatica*	0	0	0	10	0	10	20	10	0	5	10	10	10	0	100					
99	Vranjska Banja	868	41-60	*Fagus sylvatica*	0	5	0	25	15	15	10	0	0	5	0	15	30	40	100					
96	Muhovac	850	41-60	*Castanea sativa*	*										10	10	0	10	100					
60	Vrnjačka Banja	392	41-60	*Quercus frainetto*	25	20	15	20	0	10	15	0	10	10	50	60	60	60	100					
420	Gobeljska reka	1558	81-100	*Picea abies*	10	0	0	5	5	0	5	0	0	0	0	0	0	100						
420	Gobeljska reka	1558	81-100	*Abies alba*	10	0	0	10	10	5	5	0	0	5	5	5	5	100						
101	Deliblato	125	81-100	*Robinia pseudoacaccia*	75	75	75	75	60	55	50	50	30	35	75	70	80	100						
73	Pobijenik	1201	uneven aged	*Picea abies*	15	10	5	10	15	15	5	0	5	10	20	0	25	100						
42	Despotovac	386	uneven aged	*Acer campestre*	*									10	30	40	95	100						
28	Potaj Čuka	619	61-80	*Fagus sylvatica*	0	0	0	0	0	0	0	0	0	0	0	0	0	100						
24	Oreškovica	189	<= 20	*Robinia pseudoacaccia*	0	10	0	40	10	30	0	10	10	0	50	30	95	100						
21	Grošnica	591	uneven aged	*Fagus sylvatica*	*							0	15	90	85	70	60	100						
430	Vranić	165	21-40	*Ulmus minor*	*										10	85	100							
427	Kupinske Grede	70	101-120	*Fraxinus angustifolia*	15	25	25	30	20	25	45	40	35	35	75	85	100							
425	Raškovica	75	81-100	*Fraxinus angustifolia*	65	15	10	45	20	15	30	35	25	50	10	40	100							
418	Murtenica	1344	81-100	*Abies alba*	*							15	0	0	0	0	100							
418	Murtenica	1344	81-100	*Abies alba*	*							0	0	0	0	0	100							
403	Pekare	915	61-80	*Fagus sylvatica*	0	0	0	10	0	10	0	5	15	40	40	70	100							
106	Popovica	425	101-120	*Tilia platyphyllos*	5	10	0	0	0	10	20	10	5	0	0	0	100							
106	Popovica	425	101-120	*Quercus petraea*	15	15	15	15	15	20	35	20	25	15	15	15	100							
106	Popovica	425	101-120	*Fagus sylvatica*	5	5	0	5	5	10	5	5	5	0	0	0	100							
69	Bela Palanka	1355	81-100	*Fagus sylvatica*	30	30	30	40	70	80	70	90	60	0	10	0	100							
67	Makrešane	268	21-40	*Quercus frainetto*	10	10	10	10	20	10	20	30	20	30	90	90	100							
56	Lazac	383	61-80	*Quercus frainetto*	35	35	35	35	30	10	10	15	0	10	80	80	100							
21	Grošnica	591	uneven aged	*Populus tremula*	0	15	10	5	0	5	5	5	30	10	25	60	100							
415	Maljen	630	61-80	*Abies alba*	0	10	0	10	10	20	0	10	10	40	80	100								
415	Maljen	630	61-80	*Abies alba*	0	10	10	0	10	0	5	0	0	20	60	100								
415	Maljen	630	61-80	*Abies alba*	0	10	0	10	20	10	10	10	0	30	70	100								
402	Tara II	1151	81-100	*Picea abies*	30	40	10	5	10	10	0	0	0	0	5	100								
402	Tara II	1151	81-100	*Picea abies*	40	50	20	10	25	20	5	0	0	20	25	100								
402	Tara II	1151	81-100	*Picea abies*	50	55	20	10	10	10	0	0	0	15	30	100								
402	Tara II	1151	81-100	*Picea abies*	5	10	5	5	10	0	0	0	0	0	0	100								
94	Poganovo	616	41-60	*Quercus cerris*	0	0	0	40	35	40	40	20	10	90	90	100								
50	Brezova	860	81-100	*Fagus sylvatica*	5	5	0	5	30	20	10	0	0	15	80	100								
33	Bukova Glava	432	121-140	*Quercus petraea*	10	40	10	30	10	20	10	10	10	10	10	100								
23	Turija	339	41-60	*Carpinus betulus*	0	0	0	0	10	0	30	0	30	50	90	100								
15	Struganik	406	21-40	*Quercus cerris*	20	20	10	0	10	10	10	0	0	10	40	100								
15	Struganik	406	21-40	*Quercus cerris*	20	20	0	10	10	0	10	0	0	20	20	100								
15	Struganik	406	21-40	*Quercus cerris*	30	20	10	10	20	10	10	0	0	20	30	100								
14	Cer - Sever	70	41-60	*Carpinus betulus*	0	20	10	20	5	10	0	0	0	10	90	100								
7	Valjevo	268	41-60	*Quercus frainetto*	30	20	10	20	10	10	20	10	20	10	95	100								
23	Turija	339	41-60	*Acer campestre*	10	20	20	10	20	30	30	10	10	70	100									
20	Tometino Polje	632	61-80	*Betula pendula*	20	20	10	10	10	5	5	20	20	80	100									
20	Tometino Polje	632	61-80	*Betula pendula*	20	25	15	15	10	0	0	5	60	85	100									
14	Cer - Sever	70	41-60	*Carpinus betulus*	0	10	10	10	10	10	5	10	10	40	100									
28	Potaj Čuka	619	61-80	*Fagus sylvatica*	10	0	0	20	15	10	5	10	5	0	100									
56	Lazac	383	61-80	*Fagus sylvatica*	25	25	25	25	15	10	10	10	25	95	100									
69	Bela Palanka	1355	81-100	*Fagus sylvatica*	20	0	10	5	0	0	0	10	5	5	100									
415	Maljen	630	61-80	*Fagus sylvatica*	30	30	10	10	0	10	10	5	0	10	100									
415	Maljen	630	61-80	*Fagus sylvatica*	20	20	20	10	10	10	5	0	0	80	100									
20	Tometino Polje	632	61-80	*Populus tremula*	30	35	30	15	10	10	10	15	20	40	100									
20	Tometino Polje	632	61-80	*Prunus avium*	15	20	20	20	15	5	30	30	90	95	100									
15	Struganik	406	21-40	*Quercus cerris*	20	30	10	10	10	10	0	5	0	10	100									
19	Stragari	251	61-80	*Quercus cerris*	15	10	15	15	5	0	5	0	5	5	100									
19	Stragari	251	61-80	*Quercus cerris*	15	10	15	15	0	0	10	5	5	0	100									
19	Stragari	251	61-80	*Quercus cerris*	20	20	20	15	0	0	0	5	10	0	100									
19	Stragari	251	61-80	*Quercus cerris*	15	15	15	20	5	0	0	0	5	10	100									
19	Stragari	251	61-80	*Quercus cerris*	15	20	20	10	0	0	10	5	10	15	100									
29	Rudna Glava	346	21-40	*Quercus cerris*	10	20	20	20	0	0	10	10	70	95	100									
29	Rudna Glava	346	21-40	*Quercus cerris*	20	50	10	20	80	90	50	70	80	80	100									
35	Jabukovac	136	41-60	*Quercus cerris*	0	30	10	10	0	20	30	15	25	0	100									
36	Kladovo	168	41-60	*Quercus cerris*	20	40	10	20	10	0	10	20	90	95	100									
81	Kuršumlija	453	21-40	*Quercus cerris*	20	20	10	30	10	20	20	10	30	80	100									
7	Valjevo	268	41-60	*Quercus frainetto*	40	20	10	10	10	0	20	20	10	95	100									

SP Level I	Locality	Altitude (m)	Stand age	Tree Species	Defoliation (%) per year																			
					2004	2005	2006	2007	2008	2009	2010	2011	2012	2013	2014	2015	2016	2017	2018	2019	2020	2021	2022	2023
26	Ranovac	216	61-80	*Quercus frainetto*	*		10	10	30	30	10	40	50	80	100									
36	Kladovo	168	41-60	*Quercus frainetto*	10	20	20	20	0	0	20	5	20	80	100									
37	Vratarnica	231	41-60	*Quercus frainetto*	20	40	40	40	30	0	10	0	0	70	100									
37	Vratarnica	231	41-60	*Quercus frainetto*	10	10	40	20	10	0	10	0	10	0	100									
41	Rekovac	400	41-60	*Quercus frainetto*	40	40	20	30	20	20	30	30	40	15	100									
41	Rekovac	400	41-60	*Quercus frainetto*	*		0	50	60	60	50	60	95	40	100									
56	Lazac	383	61-80	*Quercus frainetto*	30	25	20	20	5	0	10	5	0	10	100									
103	Odžaci	75	<= 20	*Quercus robur*	10	5	15	15	30	35	35	30	25	95	100									
426	Grabovačko ostrvo	0	121-140	*Quercus robur*	50	55	35	20	25	35	35	75	80	80	100									
24	Oreškovica	189	<= 20	*Robinia pseudoacaccia*	10	0	10	10	0	0	0	0	20	40	100									
41	Rekovac	400	41-60	*Robinia pseudoacaccia*	20	20	15	40	70	80	80	65	60	95	100									
68	Brus	328	61-80	*Robinia pseudoacaccia*	0	0	0	90	40	40	0	60	80	90	100									
401	Tara I	1098	101-120	*Abies alba*	20	25	20	10	5	5	0	5	10	0	100									
415	Maljen	630	61-80	*Abies alba*	20	10	0	20	20	10	10	30	80	90	100									
415	Maljen	630	61-80	*Abies alba*	20	10	10	10	20	0	0	5	0	0	100									
417	Zlatar	1354	61-80	*Picea abies*	5	5	0	0	5	5	10	5	5	75	100									
59	Kraljeva kamenica	572	<= 20	*Pinus sylvestris*	15	20	15	10	0	0	0	0	15	15	100									
59	Kraljeva kamenica	572	<= 20	*Pinus sylvestris*	5	5	5	5	0	0	0	0	10	10	100									
59	Kraljeva kamenica	572	<= 20	*Pinus sylvestris*	15	15	10	5	0	0	10	0	10	10	100									
21	Grošnica	591	uneven aged	*Betula pendula*	10	10	10	5	30	30	0	0	70	100										
21	Grošnica	591	uneven aged	*Betula pendula*	15	15	15	10	0	10	0	0	90	100										
13	Povlen	1035	61-80	*Fagus sylvatica*	20	30	10	20	15	10	0	0	0	100										
21	Grošnica	591	uneven aged	*Fagus sylvatica*	10	5	0	0	0	0	0	0	10	100										
50	Brezova	860	81-100	*Fagus sylvatica*	35	30	20	20	30	0	0	0	0	100										
51	Srednja Reka	1263	81-100	*Fagus sylvatica*	*						10	80	90	100										
403	Pekare	915	61-80	*Fagus sylvatica*	0	0	0	0	0	0	10	20	85	100										
29	Rudna Glava	346	21-40	*Quercus cerris*	10	40	10	10	20	20	10	10	30	100										
100	Golemo Selo	634	121-140	*Quercus cerris*	10	10	10	40	40	40	30	10	40	100										
30	Kučevo	217	61-80	*Robinia pseudoacaccia*	10	30	30	80	40	50	70	90	90	100										
415	Maljen	630	61-80	*Abies alba*	0	20	10	20	30	10	0	80	80	100										
101	Deliblato	125	81-100	*Pinus nigra*	45	70	60	60	80	85	80	80	80	100										
40	Svetozarevo	421	41-60	*Fagus sylvatica*	0	0	0	0	10	10	5	0	0	100										
96	Muhovac	850	41-60	*Fagus sylvatica*	0	0	0	0	70	90	40	30	30	100										
409	Mali Jastrebac	569	81-100	*Fagus sylvatica*	10	10	10	10	5	10	5	15	10	100										
102	Plavna	75	<= 20	*Populus hybrides*	40	40	10	40	20	35	40	25	35	100										
102	Plavna	75	<= 20	*Populus hybrides*	*							65	60	100										
96	Muhovac	850	41-60	*Fagus sylvatica*	*				20	25	0	0	100											
21	Grošnica	591	uneven aged	*Betula pendula*	20	15	20	15	15	20	10	0	100											
23	Turija	339	41-60	*Fraxinus ornus*	10	20	20	10	20	95	20	30	100											
60	Vrnjačka Banja	392	41-60	*Quercus cerris*	15	20	10	15	0	10	10	20	100											
17	Srezojevci	554	41-60	*Quercus frainetto*	25	15	15	40	40	70	5	0	100											
23	Turija	339	41-60	*Carpinus betulus*	10	20	10	10	30	80	90	90	100											
51	Srednja Reka	1263	81-100	*Fagus sylvatica*	*						10	10	100											
95	Topli Do	1230	41-60	*Fagus sylvatica*	20	0	0	80	90	90	90	100												
64	Mozgovo	685	61-80	*Quercus petraea*	0	0	10	20	20	20	70	100												
96	Muhovac	850	41-60	*Castanea sativa*	0	0	5	20	20	30	0	100												
40	Svetozarevo	421	41-60	*Fagus sylvatica*	0	0	0	0	90	90	80	100												
48	Požega	455	61-80	*Fagus sylvatica*	35	30	5	15	0	50	50	100												
421	Vršački Breg	370	61-80	*Fraxinus ornus*	0	0	0	10	10	25	100													
75	Prijepolje	1050	41-60	*Quercus petraea*	60	70	30	30	40	45	100													
20	Tometino Polje	632	61-80	*Betula pendula*	15	20	5	10	25	15	100													
102	Plavna	75	<= 20	*Populus hybrides*	35	30	40	30	25	15	100													
102	Plavna	75	<= 20	*Populus hybrides*	15	25	10	35	15	10	100													
18	Spomen Park KG	256	81-100	*Quercus frainetto*	25	35	30	30	20	15	100													
27	Osanica	652	41-60	*Quercus petraea*	20	20	20	40	35	35	100													
105	Čortanovačka šuma	175	61-80	*Tilia platyphyllosc*	15	10	10	20	10	5	100													
23	Turija	339	41-60	*Carpinus betulus*	60	50	50	40	90	100														
416	Petkovica	214	101-120	*Quercus frainetto*	40	60	50	30	20	100														
102	Plavna	75	<= 20	*Populus hybrides*	80	80	80	80	70	100														
412	Tisovac	1145	41-60	*Fagus sylvatica*	10	0	20	10	0	100														
426	Grabovačko ostrvo	0	121-140	*Quercus robur*	65	65	65	80	100															
427	Kupinske Grede	70	101-120	*Quercus robur*	40	45	45	90	100															
37	Vratarnica	231	41-60	*Quercus frainetto*	10	40	50	70	100															
427	Kupinske Grede	70	101-120	*Quercus robur*	50	55	55	65	100															
106	Popovica	425	101-120	*Quercus petraea*	20	20	15	100																
427	Kupinske Grede	70	101-120	*Quercus robur*	65	70	90	100																
418	Murtenica	1344	81-100	*Abies alba*	10	10	20	100																
427	Kupinske Grede	70	101-120	*Quercus robur*	65	75	100																	
21	Grošnica	591	uneven aged	*Quercus cerris*	35	40	100																	
418	Murtenica	1344	81-100	*Abies alba*	20	20	100																	

SP Level I	Locality	Altitude (m)	Stand age	Tree Species	Defoliation (%) per year																			
					**2004**	**2005**	**2006**	**2007**	**2008**	**2009**	**2010**	**2011**	**2012**	**2013**	**2014**	**2015**	**2016**	**2017**	**2018**	**2019**	**2020**	**2021**	**2022**	**2023**
73	Pobijenik	1201	uneven aged	*Abies alba*	25	25	100																	
48	Požega	455	61-80	*Fagus sylvatica*	5	5	100																	
423	Kolut Kozara	70	121-140	*Quercus robur*	90	90	100																	
105	Čortanovačka šuma	175	61-80	*Tilia platyphyllosc*	65	100																		
41	Rekovac	400	41-60	*Robinia pseudoacaccia*	60	100																		

* A new tree has been singled out at this site and its monitoring has begun.

Table S2. Analysis of the most endangered tree species due to the impact of drought.

Tree Species	*Fagus sylvatica*	*Quercus cerris*	*Quercus petraea*	*Quercus frainetto*	*Carpinus betulus*	*Picea abies*	*Pinus nigra*	*Pinus sylvestris*	*Abies alba*	Other broadleaves	Total
Average No. of trees 2004-2023	846	520	185	386	115	145	60	69	67	500	2892
Participation in the total sample (%)	29.2	18.0	6.4	13.3	4.0	5.0	2.1	2.4	2.3	17.3	100
Total No. of dead trees 2004-2023	40	26	12	23	7	20	3	5	15	61	212
No. of dead trees after drought *	20	18	3	14	4	14	3	4	10	27	117

* No. of dead trees in the period of registered increased mortality (2013-2016 and 2023)

Table S3. Random yield due to the impact of drought for larger groups of trees within Forest Estates (FE) Managed by Public Enterprise (PE) “Srbijašume”.

Year	Rrandom yield of larger tree groups at the stand level (m^3)																	Total (m^3)
	**12**	**30**	**31**	**32**	**33**	**34**	**35**	**36**	**37**	**38**	**39**	**40**	**41**	**42**	**43**	**44**	**45**	
2013	691	56	0	0	174	654	1.014	4.904	2.188	721	12.284	4.076	646	680	54	470	530	29.142
2014	1.703	788	41	507	1.412	2.786	1.955	3.845	13.040	1.592	12.771	8.419	441	3.531	512	984	556	54.883
2015	1.180	77	26.841	51	1.163	1.410	1.753	1.749	10.346	2.631	2.456	5.942	7.965	2.859	472	2.136	3.718	72.750
2016	1.417	153	0	137	1.243	5.427	2.026	4.321	19.808	1.107	9.225	3.640	7.871	7.604	292	931	79.964	145.169
2017	755	82	0	166	1.111	941	1.697	6.314	18.560	179	9.568	5.896	5.239	3.942	1.750	1.032	725	57.956
2018	1.227	72	313	17	810	1.180	1.187	3.863	17.098	509	2.781	4.616	4.241	146	2.387	924	2.557	43.927
2019	947	0	0	113	1.142	2.556	1.213	2.049	15.707	312	3.505	3.158	2.229	1.075	1.929	661	7.612	44.208
2020	573	30	0	28	753	640	1.571	3.319	8.232	69	3.653	2.726	937	655	431	365	283	24.268
2021	578	76	0	219	497	600	991	1.742	541	235	3.689	376	648	334	94	314	157	11.093
2022	348	1.079	92.780	44	72	512	680	1.951	2.405	311	679	1.614	90	47	350	100	217	103.280
2023	505	0	45.697	28	45	443	819	5.940	3.668	164	967	984	145	877	109	376	47.80	60.768

12 - FE "Beograd", **30** - FE "Severni Kučaj", **31** - FE "Timočke šume", **32** - FE "Južni Kučaj", **33** - FE "Kragujevac", **34** - FE "Boranja", **35** - FE "Užice", **36** - FE "Prijepolje", **37** - FE "Golija", **38** - FE "Šumarstvo", **39** - FE "Stolovi", **40** - FE "Rasina", **41** - FE "Toplica", **42** - FE "Niš", **43** - FE "Pirot", **44** - FE "Šuma", **45** - FE "Vranje”

Table S4. Tests of Normality

		Kolmogorov-Smirnov[a]			Shapiro-Wilk		
	Years of Observation	Statistic	df	Sig.	Statistic	df	Sig.
Mortality rates	2004-2008	.135	5	.200*	.999	5	.999
	2009-2013	.309	5	.135	.829	5	.137
	2014-2018	.353	5	.041	.755	5	.033
	2019-2023	.406	5	.007	.671	5	.005

* This is a lower bound of the true significance

a Lilliefors Significance Correction
